# Does a school snack make a difference? An evaluation of the World Food Programme emergency school feeding programme in Lebanon among Lebanese and Syrian refugee children

**DOI:** 10.1017/S1368980022000362

**Published:** 2022-06

**Authors:** Zeina Jamaluddine, Chaza Akik, Gloria Safadi, Sara Abou Fakher, Nehmat El-Helou, Soha Moussa, Dominique Anid, Hala Ghattas

**Affiliations:** 1 Center for Research on Population and Health, Faculty of Health Sciences, American University of Beirut, PO Box 11-0236/EPHD, Riad El-Solh, Beirut 1107 2020, Lebanon; 2 London School of Hygiene and Tropical Medicine, London, UK; 3 World Food Programme, Beirut, Lebanon

**Keywords:** School feeding programme, Diet diversity, School attendance, School engagement, Quasi-experimental, School children

## Abstract

**Objective:**

To investigate the effects of providing a daily healthy school snack on children’s nutritional, social and educational outcomes and explore stakeholders’ perceptions of an emergency school feeding programme (SFP).

**Design:**

Convergence triangulation mixed-methods study design. Associations were examined between receiving the school feeding intervention and children’s outcomes using multivariable regression models. Quantitative data were complemented with interviews and focus group discussions with parents and staff.

**Setting:**

In vulnerable communities in Lebanon, the World Food Programme has implemented an emergency SFP targeting Lebanese (attending morning sessions) and Syrian refugee children (attending afternoon sessions) in public schools.

**Participants:**

Children from ten intervention schools (morning *n* 403; afternoon *n* 379) and ten matched control schools (morning *n* 399; afternoon *n* 401), as well as twenty-nine parents and twenty-two school staff members.

**Results:**

Diet diversity was higher in intervention schools as compared with control with a significantly higher consumption of dairy products, nuts and fruit in both sessions. Child-reported food insecurity experience was lower in children attending the afternoon session of intervention *v*. control schools. The SFP intervention was associated with higher school engagement and sense of school community in the morning session only. While the SFP was significantly associated with higher attendance for children in afternoon sessions only, it was significantly associated with school retention of children in both sessions.

**Conclusions:**

A daily healthy snack potentially acts as an incentive to improve children’s nutritional outcomes, school engagement, sense of belonging, equality between students and improvement in children’s attendance and retention in public schools.

Currently, hosting around 1·5 million Syrian refugees, Lebanon still has the highest per capita refugee concentration in the world^(1,2)^. Lebanese and Syrian refugee children attend the same public schools as part of a national response plan to ensure high educational coverage in vulnerable school aged children^(3)^. This was made possible by creating two academic sessions during the school day: a morning session for Lebanese children and an afternoon session for Syrian children^(3)^. As a result, Lebanese children’s enrolment rates in public schools remained high^(3)^, while the enrolment of Syrian refugee children aged 6 to 14 years was around 69 %^(4)^.

From a nutritional standpoint, comparable data on Lebanese and Syrian refugee schoolchildren do not exist; however, Lebanon shows signs of a ‘double burden of malnutrition’^(5)^. At one end of the spectrum, the prevalence of overweight in Lebanese children has been increasing in recent years, with the adoption of unhealthy eating patterns^(6,7)^, and child overweight was also on the rise in Syria pre-conflict^(8)^. At the other end of the spectrum, the prevalence of food insecurity remains high among Syrian refugee children along with a reduction in diet diversity^(4)^. Syrian refugees often resort to negative coping strategies by reducing food consumption and sending their children (mainly boys) to work^(9)^. There are also preliminary reports of a rise in food insecurity amongst Lebanese populations^(3,10)^.

Lebanon has recently experienced a deteriorating economic situation, coupled with profound political instability. With the COVID-19-related lockdown measures and the loss of approximately 80 % of the national currency value, vulnerable Lebanese families and refugees have been pushed further into poverty^(11)^. Recent surveys are indicating a rapid increase in food insecurity among Lebanese, 19 % were skipping a meal in May 2020 whereas 29 % skipped a meal in August 2020^(10,12)^.

In 2016, the World Food Programme (WFP) launched an emergency school feeding programme (SFP), in alignment with its standard global school feeding practices^(12)^, as part of its support to Lebanon^(13)^. This emergency SFP is provided to Lebanese and Syrian refugee children attending primary public schools (Grades 1–6) in the most vulnerable communities across Lebanon^(15,16)^.

Despite mixed evidence, ample studies assessing the effects of SFP found that those programmes have small beneficial effects on diet diversity^(17)^, weight and height^(18)^, school attendance^(19)^ and school performance^(20)^ in school children. Dietary benefits of SFP can range from the alleviation of short-term hunger to the fulfilment of essential gaps in children’s micronutrient and protein intake by increasing diet diversity^(21–23)^. Moreover, several studies found that SFP are one of the few child-centred interventions to have a positive impact on school-related outcomes^(24)^. A school snack or meal can potentially act as an incentive for parents to send their children to school that ultimately translates to higher enrolment, better attendance and improved learning outcomes^(25)^. Improvements in psycho-emotional/social well-being among children receiving school feeding have also been widely reported^(24,26)^. Literature highlights that feeding programmes can be viewed as social interventions that indirectly foster a sense of engagement, motivation, interaction and involvement within and between children^(26,27)^.

The WFP emergency SFP provides a daily healthy snack pack (fruit, nuts or dairy product) and is hypothesised to increase diet diversity and act as an incentive to improve children’s school enrolment, attendance and academic retention^(28)^. Since the beginning of the implementation of the emergency SFP in Lebanon, regular post-distribution monitoring has been conducted; however, the programme’s effectiveness at achieving its goals has yet to be assessed.

Considering the emerging crisis, a formal evaluation was commissioned in 2019. The current study aims to investigate the effects of providing a daily school snack on children’s nutritional, social and educational outcomes and to explore perceptions of stakeholders towards the emergency SFP and its potential effects. It is the first to assess outcomes in both national and refugee children, and to highlight lessons learned from the implementation of this emergency SFP, with implications on advocacy for the scale-up of these programmes.

## Methods

The current study adopted the convergence triangulation design to mixing methods, where we aimed to obtain complementary data on the same topic. This approach allowed us to compare and contrast the quantitative statistical results with qualitative findings as well as validate or enrich some of the quantitative results with qualitative findings^(29)^.

The study involved a quasi-experimental component that collected quantitative data from children attending emergency SFP (intervention) schools and matched non-emergency SFP (control) schools to assess the difference in various nutritional, social and educational outcomes between children attending intervention and control schools. This was complemented with qualitative data collection in intervention schools, including interviews with school directors and focus group discussions (FGD) with parents, teachers and school staff. The qualitative methods explored perceptions of stakeholders towards the emergency SFP and its potential effects.

### Quantitative child-level data collection

#### Study design and sample size

Prior to the current study, and on the basis of a vulnerability assessment, the Ministry of Education and Higher Education (MEHE) identified 167 schools in the most vulnerable areas in Lebanon that could receive the emergency SFP^(16)^. MEHE then randomly selected thirty-nine schools to receive the SFP. These schools received the emergency SFP provided by WFP for at least one school year. The supplementary material outlines the nutritional composition of the snack provided (see online supplemental Table S1) and a detailed flow diagram of the study’s methodology (see online supplemental Fig. S1). Pre-intervention baseline data were not available on outcomes of interest.

We designed a quasi-experimental evaluation powered to detect a difference in dietary diversity between children attending control and intervention schools. Power calculations were conducted pre-hoc as part of the evaluation study. The study was powered to enable a stratified analysis by refugee status (Lebanese children attending the morning session and Syrian refugee children attending the afternoon session) and detect differences in each stratum. Taking the cluster design into account, to detect a difference of 1 point in dietary diversity score, with a SD of 1·2, intra-class correlation of 0·5, with 95 % confidence and 80 % statistical power, the required sample size was found to be eighty students per school (forty children in the morning session and forty children in the afternoon session), attending twelve control and twelve intervention schools.

In order to select the study sample, we matched each of the thirty-nine intervention schools (list provided by the MEHE) to a ‘closest match’ school from the 128 non-programme schools. MEHE provided us with GPS locations of the thirty-nine intervention schools and 128 possible controls. In Lebanon, public schools in close proximity to each other have similar catchment areas and cater to families of similar socio-economic backgrounds. In collaboration with MEHE, each intervention school was matched to a control school based on specific school characteristics, i.e. 10 km radius, size of the school, school type (co-ed *v*. single-sex schools), other interventions occurring in the schools and whether the schools operated an afternoon session. The matched schools were evaluated using propensity scores to determine the closest matched control school for each of the thirty-nine intervention schools.We randomly selected twelve matched pairs of schools (twelve intervention and twelve control) using a systematic random sample listing the thirty-nine matched pairs of schools from the smallest to the largest.

Data collection began on 30 January 2020 and was planned to continue through 4 March 2020; however, due to the forced school closures that came into effect on 28 February 2020, in response to the emerging COVID-19 pandemic, we were unable to complete the quantitative data collection in two control schools and two intervention schools. The final sample was composed of twenty schools (ten control schools and ten intervention schools).

#### Recruitment

After obtaining approval from MEHE, school directors were contacted by telephone, whereby an explanation of the study was provided and approval to conduct the evaluation was sought. Within intervention and control schools, the two strata of Lebanese children (morning session) and Syrian refugee children (afternoon session) were randomly sampled.

In the twenty recruited schools (ten control schools and ten intervention schools), fifty parent/caregiver consent forms were distributed per grade (grades 4, 5 and 6) in all schools in both morning and afternoon sessions (totalling 150 per school session and 6000 overall). Consent forms and a short parent socio-demographic questionnaire were sent home to parents/caregivers, and children were asked to return the signed forms in sealed envelopes. The parental response rate (children whose parents consented to participate in the study) for the morning session was around 80 %, while the response rate for the afternoon session was around 93 % (see online supplemental Fig. S1).

A total of forty children in the morning session and forty children in the afternoon session were randomly selected from the total pool of children whose parents/caregivers provided consent in order to participate in the study. All selected children received verbal information pertinent to the purpose and the overall course of the survey. Child assent was consequently obtained from all children in private preceding survey administration. None of the children refused to participate in the survey.

#### Outcomes

The parent socio-demographic questionnaire was self-administered and included sex and employment of the head of the household and the highest level of education of the mother/main female caretaker (never attended school, basic education, intermediate education, secondary school and university degree).

The child questionnaire included several modules, including a diet recall, a food security module and a social well-being module. In addition to the survey, we collected data on educational outcomes from school records.

#### Nutritional outcomes

A qualitative diet recall questionnaire, which talks the child through each meal consumed in the last 24 h, was used to assess short-term hunger and diet diversity. To facilitate and ensure proper child recall, the day was divided into meals and locations (e.g. breakfast, on the way to school, at school, lunch, snacks and dinner). Data collectors were extensively trained by dietitians on how to effectively administer the dietary recall. Accordingly, a dietary diversity score (DDS), defined as the number of food groups consumed over the past day, was calculated. For this, the diet was classified according to eleven food groups, as recommended by FAO, which include (1) cereals, roots and tubers; (2) vitamin-A-rich fruits and vegetables; (3) green leafy vegetables; (4) other fruits; (5) other vegetables; (6) legumes; (7) nuts; (8) meats, poultry and fish; (9) fats and oils; (10) milk and dairy products and (11) eggs^(21,30)^. DDS has been validated for several age/sex groups as proxy measures for macro and/or micronutrient adequacy of the diet^(31)^. This score is an appropriate method to evaluate nutrient intake adequacy in infants, young children and adolescents^(30)^. With the aim of further assessing children’s diets, we classified foods into four additional groups including salted snacks, sweets, sweetened beverages and zaatar (a mix of herbs/spices including dried oregano, thyme and sesame seeds commonly consumed for breakfast); these groups were not included in the dietary diversity scoring but rather interpreted as stand-alone groups.

Child food security was examined using a child food security questionnaire previously validated in this context and study age group^(32)^. This approach has recently been shown to be an accurate measure of a child’s experience with food insecurity^(33,34)^. For all children, a food insecurity experience score was generated using ten items. Children were classified as food secure (scores 0–2) or food insecure (scores 3–10)^(32)^.

#### Social outcomes

A school engagement scale, a set of sense of school community questions and a self-esteem scale^(35–37)^, all derived from validated tools for school aged children, were included to examine the association between the programme and school engagement and self-esteem. Children’s school engagement was analysed according to three domains: behavioural, emotional and cognitive. In order to analyse the sense of school community, children were presented with a set of four statements. Children were asked to describe to what degree they experience/agree with the presented statements, data were analysed accordingly. A self-esteem score, out of 30, was calculated for each child^(36)^.

#### Educational outcomes

Data on school absences were collected from school records as the number of missed days within the previous academic year (months October 2018 to May 2019) for all children in grades 3, 4 and 5 (as the programme was targeting primary school children). We considered a child’s total absenteeism as the sum of total days absent during a school year. We report on absenteeism categorised as the attendance of 70 %, 80 % and 90 % of school days in the academic year. From the records, we also collected data related to child dropout as the number of months missed due to abrupt termination of scholastic enrolment from each school.

#### Statistical analysis

We assessed differences in schoolchildren’s nutritional, food security, school engagement, sense of community, self-esteem, school dropout and absenteeism outcomes between children attending intervention schools and children attending control schools. Results of the morning and afternoon sessions are presented separately.

We conducted multivariable linear and logistic regression analyses to examine the associations between programme participation and outcomes, controlling for covariates. Models were adjusted for child sex, age, school location and gender and employment of the head of household. The results were adjusted for clustering by school. School absenteeism was analysed using negative binominal regression models adjusting for school location and size and student grade-level. A *P*-value of 0·05 was used to indicate statistical significance. All analyses were performed using Stata 15 (StataCorp.).

### Qualitative school-level data collection

Qualitative data collection involved interviews with school directors and FGD with parents and school staff including teachers, health coordinators and floor supervisors. Qualitative data collection was completed before COVID-19 school closures.

#### Recruitment

##### Interviews with school directors

The twelve directors of intervention schools (seven women and five men) were approached directly, and consent to participate in the study was obtained in private. Twelve face-to-face semi-structured interviews were conducted covering topics such as programme design and delivery, snack acceptance, implementation challenges and recommendations to improve the programme.

##### Focus group discussions with parents and school staff

Parents and school staff – specifically math, biology and civic education teachers of grades 4, 5, 6 as well as the floor supervisor and health coordinator at six schools received a general information sheet, whereby they were informed of the purpose of the FGD. Parents and staff were asked to indicate their interest in participating in the study by providing their phone numbers on the general information sheet and returning the slip in a sealed envelope to the research team if they wished to participate in the FGD. The research team contacted interested participants to arrange the times of the FGD. A total of three FGD with teachers and three FGD with parents were conducted. The six FGD were conducted with a total of twenty-nine parents and twenty-two school staff participants who gave written consent to participate. The FGD took place in different geographic areas in Lebanon, and with Lebanese and Syrian parents. FGD guides with parents and school staff covered topics related to the school’s experience with the emergency SFP, interactions with/support from communities, as well as perceived benefits for children, schools and communities. Parents and school staff were also asked about whether they perceived the snack to have an impact on school outcomes.

#### Analysis

Interviews and FGD were voice recorded after obtaining written consent and subsequently transcribed. Transcripts were analysed using thematic analysis in Dedoose software. An initial reading of transcripts allowed the development of a preliminary list of emerging themes. We consequently organised the data into categories and identified relationships among and between categories, which ultimately allowed us to understand explanatory patterns.

## Results

### Children’s characteristics

A total of 1582 children in twenty schools (ten intervention schools, ten control schools) were randomly selected to participate in the study (see online supplemental Fig. S1). Table 1 presents child and household characteristics of study participants. This table shows that control and intervention participants were balanced in each of the morning and the afternoon sessions. For the analysis below, we examine the difference between intervention and control in each session separately (stratified by session).

**Table 1:** Household and child-level characteristics of children attending the morning and afternoon sessions in control and intervention schools

		Morning session			Afternoon session		
		*n*	Control	Intervention	*n*	Control	Intervention
			%	%		%	%
Child level	Sex	802			780		
	Male		51·38	56·08		40·65	48·02
	Female		48·62	43·92		59·35	51·98
	Age	802			780		
	8–10 years		35·09	30·77		16·71	13·98
	11–13 years		57·39	62·03		67·58	69·39
	14–16 years		7·52	7·20		15·71	16·62
	Grade	802			780		
	4		35·34	38·71		33·67	39·58
	5		31·58	30·02		33·67	35·09
	6		33·08	31·27		32·67	25·33
	Nationality	802			780		
	Lebanese		87·47	84·62		0·00	1·32
	Syrian		6·77	10·17		99·50	98·15
	Palestinian		5·26	3·47		0·25	0·26
	Other		0·50	1·74		0·25	0·26
Household level	Gender of the household head	789			767		
	Male		92·93	90·84		88·97	84·24
	Female		7·07	9·16		11·03	15·76
	Employment of the household head	758			743		
	Unemployed		21·41	28·00		54·52	59·83
	Employed Part Time		44·39	38·40		31·52	28·93
	Employed Full Time		34·2	33·60		13·95	11·24
	Highest level of education of the mother/main caretaker	708			678		
	Never Attended School/Basic		48·2	50·14		60·58	60·06
	Intermediate		28·53	28·24		28·70	27·33
	Secondary School Baccalaureate degree		12·19	14·99		9·28	10·81
	University		11·08	6·63		1·45	1·80
	Governorate	802			780		
	Akkar North		30·33	28·29		29·93	28·50
	Bekaa Baalbek		30·58	31·51		30·17	31·66
	Mount Lebanon		19·55	21·34		19·95	18·47
	South		19·55	18·86		19·95	21·37

The morning session sample had a mean age of 11 years (11·23 ± 0·07 years in the control and 11·23 ± 0·67 years in the intervention group), most children were of Lebanese nationality with other children being of either Syrian or Palestinian origin (residing in Lebanon-before the Syrian crisis) (Table 1). Almost all children in the afternoon session were of Syrian nationality with a mean age of 12 years (12·04 ± 0·08 years in control and 12·01 ±0·07 years in intervention group) (Table 1).

In terms of household characteristics, nearly all children in the morning session came from male-headed households, most of whom were employed. Around half of their main female caretakers reported never attending or not completing primary school (Table 1). Similarly, children in the afternoon session were found to come mostly from male-headed households, most of whom were unemployed (Table 1). Roughly 60 % of mothers reported never attending or not completing primary school (Table 1).

### Emergency school feeding programme and children’s nutritional outcomes

#### Dietary habits

**Fig. 1:**
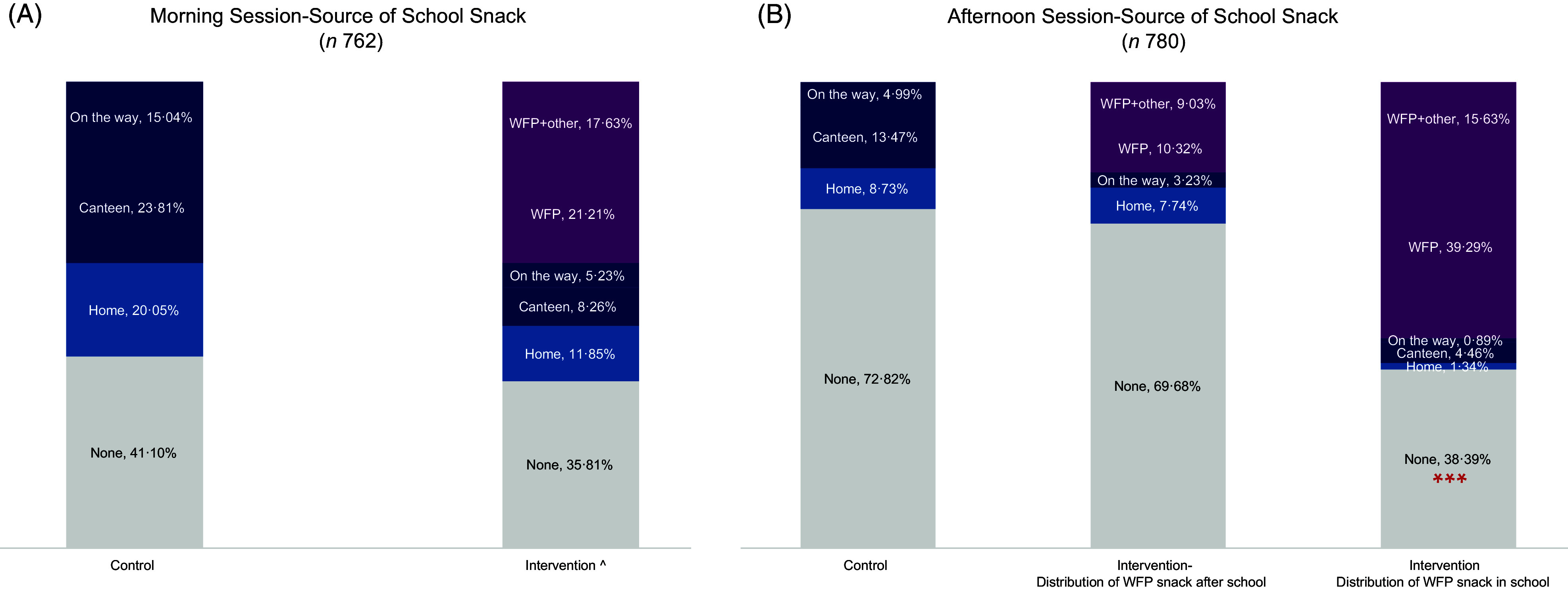
Source of school snack for children attending both morning and afternoon sessions. ^ Intervention schools that distribute snack in school, ***P < 0·01. (A, B) 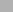, None; 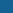, Home; 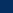, Canteen; 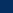, On the way; 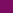,WFP; 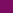, WFP + Other

In the morning session, we observe no association between children’s dietary habits and snack distribution, meaning that in both control and intervention schools, 35–40 % of children do not eat anything at school, even in the presence of a free school snack (Fig. 1(a)). Although a 5-percentage point difference between intervention and control school was noted, this difference was not statistically significant (Fig. 1(a)). While the main source of snacks in the control school was from the school canteen, the child’s home and surrounding neighbourhoods of the school were also prominent snack sources. In the intervention schools, on the other hand, the WFP snack was the main source of food consumed at school (Fig. 1(a)). Children who did not eat the snack at school took it home and a total 98 % of children consumed the snack at school or home in the previous school day (data not shown). ‘On the way’ in Fig. 1(a) represents the snack intake while walking/driving to and from the school.

The dietary habits of children attending the afternoon session in intervention schools were different from the children in control schools. The differences observed might be the result of not only snack provision but rather snack distribution timing. In fact, school director interviews highlighted that four schools out of ten were distributing the school snack after school hours rather than during the afternoon session school recess. In the intervention schools that distribute the snack before recess, a significantly higher proportion of children consumed a snack at school (62 %) as compared with children attending control schools (28 % intake of snack) (*P* < 0·001) (Fig. 1(b)). Whereas children attending intervention schools that distribute the snack after scheduled school hours demonstrate a similar snack-intake pattern as children attending control schools (31 % *v*. 28 % school snack intake in school, respectively) (Fig. 1(b)).

In the afternoon session, children receiving the snack at the end of the school day, either stayed at school and consumed the snack on school premises or consumed it while walking/being driven home (referred to as ‘on the way’) or consumed it at home (Fig. 1(b)).

School directors and staff reported two major reasons for the delayed snack distribution: (1) during the first year of the emergency SFP implementation, some schoolchildren in both shifts discarded the snack as it was not to their taste, mainly milk, as there is a cultural preference to consume it warm or with sugar or other foods, which led the school management to adopt this practice to reduce waste and (2) one school director reported that the large numbers of students in their afternoon session (almost four times those in the morning session) made it logistically impossible for the snack to be distributed prior to or during the 15-min recess:

We tried to distribute the snack during the break, but it was not effective and we tried distributing the snack during classes and it was not practical. There are more than 400 students in the afternoon [session].

#### Diet diversity

The mean DDS was significantly higher among children attending intervention schools in the morning and afternoon sessions compared with their respective controls (4·57 ± 0·10 in control *v*. 5·23 ± 0·14 in intervention in the morning session; 4·47 ± 0·08 in control *v*. 5·34 ± 0·11 in intervention schools in the afternoon session) (*P* < 0·001) (Table 2).

**Table 2:** Nutritional, social and educational outcomes of children attending morning and afternoon sessions in control and intervention schools

		Morning Session						Afternoon Session					
			Control		Intervention				Control		Intervention		
		*n*	Mean	se	Mean	se	*P*-value	*n*	Mean	se	Mean	se	*P*-value
Nutritional outcomes	Diet diversity												
	Diet diversity score	756	4·57	0·10	5·23	0·14	<0·001	737	4·47	0·08	5·34	0·11	<0·001
	Diet diversity score snack1	757	1·00	0·08	1·51	0·13	0·005	739	0·37	0·05	0·66	0·04	<0·001
	Diet diversity score snack2	757	0·52	0·03	0·53	0·06	0·920	739	0·32	0·04	0·84	0·07	<0·001
	Food security												
	Food security categorical												
	Food insecure (%)	723	32·15		30·72		0·741	683	57·66		43·74		<0·001
	Food security score	723	2·14	0·12	2·12	0·16	0·951	683	3·71	0·19	2·75	0·13	0·001
Social outcomes	School engagement scale												
	Behavioural engagement score	753	3·97	0·03	4·07	0·03	0·060	731	4·04	0·03	4·05	0·03	0·733
	Emotional engagement score	745	4·23	0·04	4·40	0·04	0·006	731	4·36	0·05	4·43	0·04	0·258
	Cognitive engagement score	749	3·50	0·06	3·52	0·06	0·818	731	3·49	0·05	3·42	0·05	0·383
	Sense of school community												
	Feel safe at school												
	A lot/very (%)	757	71·67		79·26		0·001	739	70·36		73·78		0·381
	Feel safe commuting to school												
	A lot/very (%)	757	55·45		61·7		0·068	739	51·85		49·54		0·325
	Like going to school												
	A lot/very (%)	757	73·44		79·41		0·211	739	82·06		83·99		0·599
	Teachers work hard to make sure I learn												
	A lot/very (%)	757	84·25		86·44		0·509	739	86·35		89·04		0·463
	Self-esteem												
	Self-esteem score	757	21·95	0·24	21·93	0·16	0·946	739	21·57	0·16	21·34	0·13	0·314
Educational outcomes	Dropout												
	Dropout (%)	2384	1·74		0·44		0·003	3011	17·77		3·36		<0·001
	Absenteeism												
	Total absenteeism (days)	2384	5·65		5·37		0·511	3011	13·09		8·14		<0·001

Models were adjusted for child sex, age, location of the school, gender of head of household, employment of head of household and clustered by school.

**Fig. 2:**
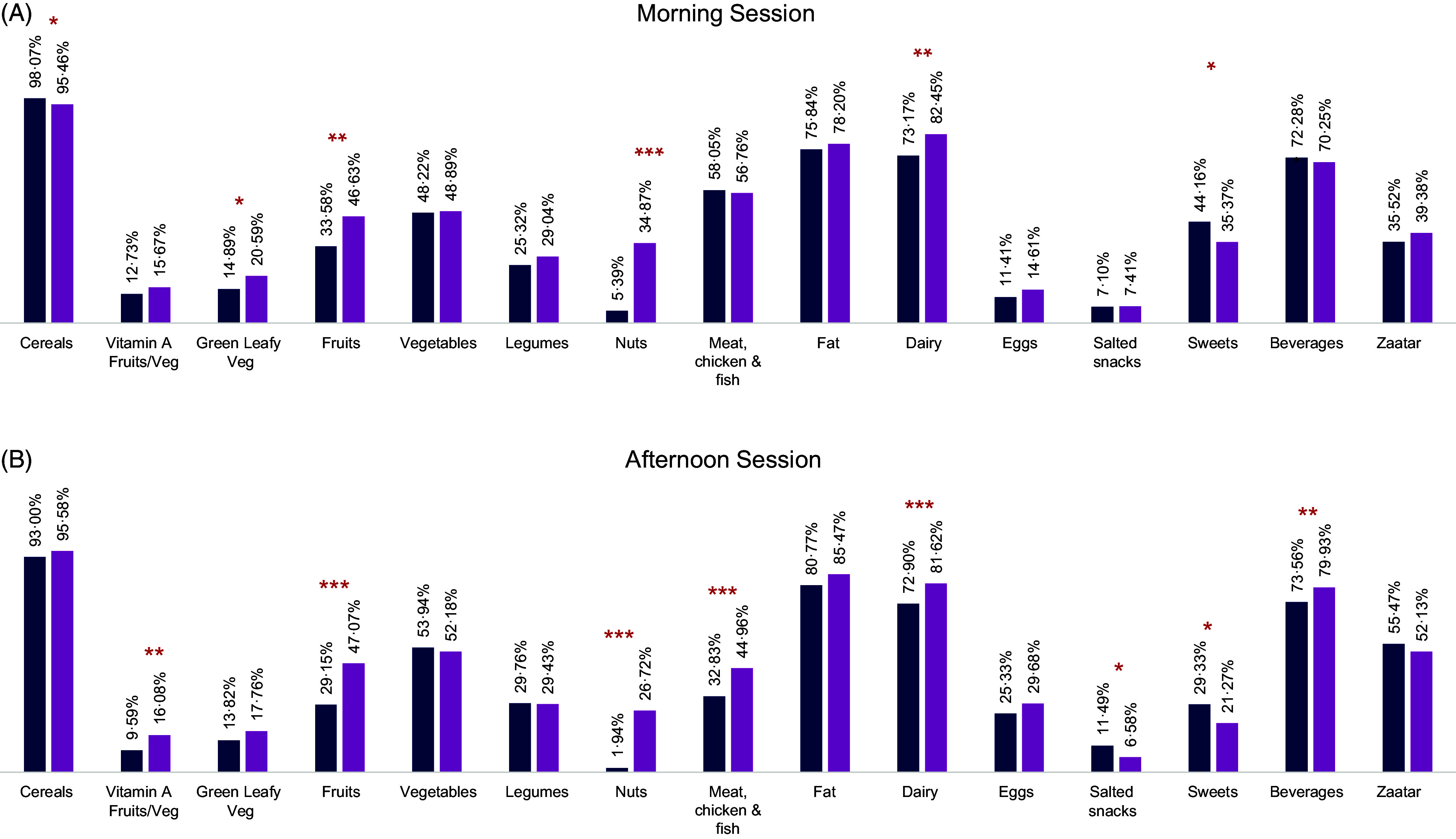
Proportion of children that consume each of the food groups, over a 24-h recall period in control and intervention schools. Models were adjusted for child age, sex, location, gender of the head of the household and household employment status (*P < 0·05, **P < 0·01, ***P < 0·001). (A, B) 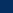, Control; 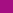, Intervention

In the morning session, this was reflected in a significantly higher intake of cereals, green leafy vegetables, fruits, nuts and dairy products and a significantly lower intake of sweets among children attending intervention schools as compared with children in control schools (Fig. 2(a)). In the afternoon session, children participating in the emergency SFP reported a significantly higher intake of vitamin A-rich fruit and vegetable, fruits, nuts, meat, dairy products, sweetened beverages and a lower intake of salted snacks and sweets as compared with control schools (Fig. 2(b)).

Discussions with school staff also revealed perceptions that the snack has increased children’s micronutrient intake and diet diversity. One staff member in a rural school noted:

Maybe they [families] do not focus a lot on fruits and vegetables. […] They might not bring home fruits especially in the current [economic] situation we are in, so, this [snack] is essential for their nutritional balance. Even the peanuts… they don’t eat nuts [at home] which are rich in minerals, so they have deficiencies. This snack covers this nutritional deficiency.

#### Food security

In the morning session, child-reported food insecurity experience did not differ significantly between children attending control (32 % food insecure) and intervention schools (30 % food insecure). However, in the afternoon session, children attending control schools were more likely to report experiencing food insecurity (57 % food insecure) as compared with children attending intervention schools (43 % food insecure) (*P* < 0·001) (Table 2). In fact, children in intervention schools reported being less likely to skip a meal (37 % control, 21 % intervention) and less likely to be hungry and not eat (30 % control, 17 % intervention).

Interviews and FGD with school directors and staff revealed geographic disparities in perception of the snack. It was perceived to substitute a meal and in turn alleviate hunger for the majority of Lebanese and Syrian students in the North and Bekaa (these are also the regions where the largest difference in the child reported food insecurity between control and intervention school in the morning session were reported – data not shown). The same applies for Syrian students in the South but not the Lebanese who bring their sandwiches and thus the snack in those cases is considered as a supplement. In Mount Lebanon, it was perceived to be a snack rather than a meal substitution for both communities.

### Emergency school feeding programme and children’s social outcomes

#### Social engagement and sense of school community

Discussions with school staff and parents revealed that students often exchange snack items with their peers, particularly milk, either because they do not like it or to collect more milk boxes to take home and prepare other dishes with. A staff member in Akkar mentioned:

A whole class once collected the milk and they made riz-b-halib [rice pudding] the next day and shared it among themselves.

This exchange has been reported to bring them together, teach them to share and be compassionate towards each other, as reported by school staff in the Bekaa:

I noticed it twice. There are children who collect them [snack items and tell their classmate]: take it, I don’t want my portion today. They are aware of this child’s social situation … They are considerate of each other’s’ feelings.

In fact, children attending the morning session in the intervention schools had significantly higher emotional (*P* < 0·01) school engagement scores and were also significantly more likely to report ‘feeling safe at school’ (*P* = 0·001) as compared with control schools (Table 2). There are no statistically significant differences in the school engagement score and sense of school community statements between children attending the control and intervention schools in the afternoon sessions.

#### Self-esteem

Several teachers, Lebanese and Syrian parents as well as some school directors indicated that the snack was perceived as giving the children a sense of self-worth:

An emotional support. They give the child his importance. I am present at school. Someone is thinking of me. Someone is supporting me.

However, quantitative data indicated no statistically significant difference in self-esteem reported by children attending intervention and control schools in both morning and afternoon sessions (Table 2).

#### Equality between children

School staff and parents in the Bekaa region revealed that the snack has promoted equality among children who may be coming from various social backgrounds. School staff in the Bekaa said:

I feel they are all equal now. Equality between students. Not all of them would have an apple. Some of them would, others not. In this way, we are all the same.

### Emergency school feeding programme and children’s educational outcomes

#### Dropout

Due to missing data and the lack of availability of some school records, we were able to match data on absenteeism to our dataset in eight intervention and eight control schools in the morning session (*n* 2384) and six intervention schools and six control schools in the afternoon session (*n* 3011). Participation in the emergency SFP was associated with a significant decrease in school dropout for children in the morning and afternoon sessions (Table 2). Interventions schools had a 0·4 % dropout rate in the morning session (*v*. 1·7 % in controls) and 3·4 % dropout rate in the afternoon session (*v*. 17·8 % in controls) (Table 2). These data indicate that schools where the intervention is taking place have higher retention of children.

#### Attendance

While in the morning session, no significant association between the emergency SFP and school absenteeism was observed (Table 2), we note that children who received the emergency SFP snack in the afternoon session were absent from school on fewer days (*P* < 0·001) than children from control schools. In the afternoon session, when absenteeism was categorised as children who attended 70 % of the school year, 85 % of the school year or 90 % of the school year, children in SFP schools had lower school absenteeism compared with control schools (*P* < 0·01, *P* < 0·01 and *P* < 0·05, respectively) (Table 2).

While many parents did not perceive that the snack affected school attendance, some Lebanese and Syrian parents and staff from one school perceived it as an encouraging factor for children to attend school explaining that they consider it as a motivation for them:

Some children do not enjoy going to school. They do not have the desire, but when the child can see that [this snack] is being distributed at school, he might go to school for this reason. – Syrian parent in the North

There is no more absence. No one is absent in morning sessions. […] So many people come only for the snack. – Staff member in the North

## Discussion

The current study shows that snack distribution through an emergency SFP is associated with higher child diet diversity and food security, with potential knock-on effects on school attendance as well as on psychosocial well-being in this context of chronic crisis^(38)^. These results are aligned with the accumulating global evidence on the impact of SFP^(39,40)^ and add contextual knowledge in a region where few SFP evaluations that highlight implementation fidelity and effectiveness have been conducted^(41)^.

The effect of the SFP in the afternoon session was larger and more significant than the effects observed in the morning session, specifically as Syrian refugees (afternoon session) started worse off than Lebanese children (morning session) with lower diet diversity, higher food insecurity and higher dropout rates.

The association between dietary habits and SFP is dependent on the timing of the snack distribution, complementary interventions provided at schools and the context. In the current study, the timing of the snack distribution was found to be essential in improving dietary habits and increasing the intake of school snacks of children attending the afternoon session, with a larger proportion of children consuming food in school when the snack is distributed before recess. This is aligned with evidence that indicates that providing a snack during school hours might alleviate short-term hunger and increase concentration and attention in schools^(42)^.

In line with our findings, several studies have also shown that SFP are associated with higher children’s diet diversity^(19,26,43–45)^. The design of the snack provided focused on improving diet diversity rather than energy intake which falls in line with recent recommendations for the composition of school meals^(23)^. Thus, in the current study, children’s diet diversity was higher in the intervention schools with a consistent significant higher consumption of the distributed food groups: dairy products, nuts and fruit. In Lebanon, a middle-income refugee-hosting country, where childhood overweight rates are high and wasting is almost negligible, the SFP ensures the availability of an alternative healthy snack at school. Recent data on Syrian refugees showed an increase in moderate and severe food insecurity among Syrian refugees from 29 % in 2019 to 49 % in 2020, associated with reduced consumption and difficult access to diversified food^(2,4)^. In our context, the snacks in afternoon sessions have helped to fill the nutritional gap and significantly improve the DDS of Syrian children.

The current study’s findings are in line with literature that has shown that SFP may indeed be associated with child’s food insecurity status – and that this is especially true for children with lower baseline socio-economic status^(46)^. In our study, a significantly lower child reported food insecurity experience was found among children attending the afternoon session in intervention schools; a population with high unemployment rates among parents, and generally high food insecurity rates^(4)^. It seems that, for poor families, the monthly value of a full meal at school could be equivalent to an average of 10 percent of their monthly income^(47)^. Promoting the development and effective implementation of food security and social protection services is crucial in the prevailing economic crisis Lebanon is currently facing. In light of our present findings, SFP may be viewed as core intervention in the management of this chronic food insecurity crisis, especially in vulnerable geographic areas (Akkar, North, Bekaa and Baalback Hermel).

The literature points to mixed results related to the association between SFP and children’s well-being along with classroom behaviour such as attention and participation. In fact, some studies report that students participating in SFP gain higher self-esteem, feel more secure, show fewer worries and are more interested in school^(48)^. On the other hand, a school breakfast programme and a school lunch programme did not have a significant effect on children’s behaviour, sense of belonging at school^(49)^ or child well-being^(50)^. Some of our data align with several studies where SFP is considered a social intervention that engages, motivates and stimulates students^(27)^, and qualitative data in our study indicate that it is perceived to improve school engagement, sense of belonging and the feeling of equality between students. In fact, similar to other studies, qualitative data indicate that this snack might promote social interaction and help children develop better relationships with their classmates^(26,48)^.

The effects of an SFP on the psycho-emotional and social well-being of students are also likely potentiated by complementary actions^(42)^. Teachers’ quality of teaching along with the school programme and environment play a key role in this matter, and this might explain the differences between morning and afternoon sessions. As indicated in the results, the emergency SFP had significantly higher school engagement and sense of school community among Lebanese but not among Syrian children. Multiple factors including legal and safety concerns as well as everyday social practices that exclude refugees might affect school engagement, social well-being and sense of community among refugee children and hinder full integration of Syrian refugees in schools^(51,52)^. This is reflective of the larger structural environment relating to the inclusiveness of Syrian refugees in Lebanon. Some evidence shows that educational spaces are places where young refugees are at times able to blur boundaries of belonging^(53)^. With that being said, it becomes crucial to prioritise school policies and services that favour social inclusivity in order to minimise the existing social gap between children and maximise the effect of school feeding programmes on all aspects of school engagement and belonging.

Multiple studies have documented the impact of SFP on access to education (enrolment, attendance)^(28)^. In fact, several studies have found that implementation of SFP in schools increases school enrolment by around 10 %^(54–56)^. Other studies found that participation in SFP is associated with better school attendance and retention, with some studies indicating an increase of 4 to 6 attendance days a year^(28,39,57)^. As per the most recent Vulnerability Assessment of Syrian Refugees, 69 % of children of primary school age (6 to 14 years old) go to school and retention rates among this population are low^(4)^. This mixed-methods assessment showed evidence of higher attendance and retention rate of Syrian refugees in schools receiving the SFP. It is important to keep in mind that this higher attendance and retention could be attributed to several factors. It may be that the SFP plays a role in improving the reputation of schools, thus contributing to higher enrolment and attendance. Most interesting, we note that this intervention increases school retention of children in both morning and afternoon sessions, with a larger difference in the latter. As noted above, Syrian refugees experience higher rates of food insecurity, therefore providing a snack could constitute a sufficient incentive to increase retention in emergency SFP schools.

### Limitations

One key limitation of the current study is the absence of data from baseline (pre-distribution of snacks) in control and intervention schools. With the absence of baseline data on outcomes of interest, it is impossible to determine whether the control and intervention groups were comparable at baseline. We were unable to establish pre-intervention exposure conditions and conduct a difference in difference analysis. By matching the control and intervention school using school-level characteristics, the aim was to try to get as close a match as possible, knowing that it is likely that parents from the same region who send children to neighbouring public schools have similar socio-economic status. In addition, although the study had a relatively high response rate (81 % and 93 % of morning and afternoon session children), we do not have any data on children whose parents refused for their children to participate in the study to assess whether there were significant socio-demographic differences between those who consented and those who did not, this may limit the generalisability of the current study.

Due to COVID-19, we were unable to collect data from four schools; however, the sample size calculation was based on detecting a 1-point difference in DDS which the current sample size was able to detect. The impact of the sample size reduction could be that we did not detect small effect sizes in other outcomes.

In terms of school absenteeism, some schools did not keep records of child attendance from previous years or had inconsistent data recording systems that we could not rely on. This could limit the interpretation of the school absenteeism and dropout results as they may be specific to schools that kept better records.

Another limitation is the lack of information on whether children who dropped out of schools were true dropouts or transfers to other schools. Regardless, retention in emergency SFP schools was higher than in control schools.

## Conclusion

The WFP emergency SFP in Lebanon is associated with notable improvements in diet diversity among both Lebanese and Syrian children and with significant reductions in reported food insecurity experience of Syrian refugee children. The school snack was additionally perceived to be associated with improved social cohesion and was perceived to instil equality between children attending the morning session. It was associated with significantly higher school retention and lower absenteeism among Syrian children attending afternoon school sessions and therefore has an important role to play in the MEHE strategy. However, in some schools, especially in the afternoon session, the delay in distributing the snack until the end of the day raises questions regarding programme fidelity, with implications on snack consumption, and a potential reduction in nutritional and educational benefits for children.

In conclusion, with the current changes in food security situation of Lebanon and the expected increase in poverty rates, expansion and proper implementation of the programme has the potential to improve diet diversity, food security as well as increase retention in schools.

## Supplementary material

For supplementary material accompanying this paper visit https://doi.org/10.1017/S1368980022000362. click here to view supplementary material
