# Supplementary material for: Does a school snack make a difference? An evaluation of the World Food Programme emergency school feeding programme in Lebanon among Lebanese and Syrian refugee children
Source: Public Health Nutr. 2022 Feb 14;25(6):1678–90. doi: 10.1017/S1368980022000362 (PMC9991733; doi:10.1017/S1368980022000362)
Supplement: Supplementary file 1 [file S1368980022000362sup001.docx]

Does a school snack make a difference? An evaluation of the World Food Programme Emergency School Feeding programme in Lebanon among Lebanese and Syrian refugee children.

# **Online Supplementary- School snack assessment**

The nutritional composition of the emergency SFP snack was analysed to assess and evaluate its overall nutritional content. For the purpose of this analysis, we refer to the month of January (a representative month), within WFP’s 2019-2020 school feeding programme calendar, as the distribution-frequency reference point. In this month, carrots were distributed on 5 days, bananas and apples on 7 days, milk on 9 days and peanuts on 10 days. Based on this, an average daily nutrient contribution provided by the snack was computed. This was achieved by multiplying each food item’s nutrient profile, derived from US Department of Agriculture (USDA) databases, by its respective monthly distribution frequency divided by the number of school days per month. The obtained estimate of the snack’s daily nutrient composition was then compared against the DRIs [1].

When compared against the DRIs, the snack meets around 13% of children’s’ caloric needs per day. The snack additionally meets 7.5% of protein needs, 18% of fat needs, and 14.4% of carbohydrate of children’s needs. The snack is also rich in vitamins and minerals and covers around 51% of vitamin A, 21% of vitamin C and 12% of folate of children’s needs per day.

In the morning session, the most consumed snacks in the intervention schools were apples, nuts, milk, bread and zaatar, whereas in the control schools, unhealthy snacks were mostly consumed at school (chocolate, boxed juice, bread, manakeesh, zaatar). Similarly, in the afternoon session, the most consumed snacks in the intervention schools were nuts, milk, apples, bananas, and carrots, whereas in the control schools mainly unhealthy foods were consumed (cookies, chips, apples, chocolate, boxed juice).

Table S1- NUTRIENT COMPOSITION OF SNACKS COMPARED TO THE DIETARY REFERENCE INTAKES (DRI)

| **Nutrient** | **Average Snack per day** | **Recommended DRI ^1^** | **% DRI met from the snack** |
| --- | --- | --- | --- |
| **Calories (kcal)** | 230 | 1700^ | 13.5 |
| **Protein (g)** | 6.4 | 85 | 7.5 |
| **Fat (g)** | 10.2 | 57 | 18 |
| **Carbohydrates (g)** | 30.6 | 212 | 14.4 |
| **Sodium (mg)** | 148.3 | 2200 | 6.7 |
| **Iron (mg)** | 0.6 | 8 | 7.5 |
| **Folate (mcg)** | 36.6 | 300 | 12.2 |
| **Zinc (mg)** | 0.8 | 8 | 10 |
| **Calcium (mg)** | 97.8 | 1300 | 7.5 |
| **Vitamin A (mcg)** | 306 | 600 | 51 |
| **Vitamin D (mcg)** | 0.8 | 15 | 5.3 |
| **Vitamin C (mg)** | 9.5 | 45 | 21.1 |

*Source: (^1^): Dietary Guidelines for Americans 2015-2020 Eight Edition for children aged 9 to 13 years both males and females. (^): Corresponds to the mid-range recommended intake for males and females.*

# **Online Supplementary-Figure S1- Flow diagram of study methodology**

167 elementary-level-schools present in the most vulnerable areas in Lebanon

39 Emergency SFP (intervention) schools

128 not receiving emergency SFP (potential control) schools

We matched each of the 39 intervention schools to a comparable non-programme school, to obtain the “closest match”. From the 39 schools that were paired we randomly selected 12 paired schools using a systematic random sample listing the schools from the smallest to the largest.

**Non-emergency SFP (control)- 12 schools**

**Emergency SFP (intervention)- 12 schools**

Due to the forced school closures that came into effect in response to the emerging COVID19 pandemic, we were unable to complete the quantitative data collection in 2 control schools and 2 intervention schools.

**Emergency SFP (intervention)**

**Parent consent forms sent** 10 schools n= 3000

- Morning session n=1500 (n=150 per school)
- Afternoon session n=1500 (n=150 per school)

Based on sample size calculation, random selection of

In 10 schools n=800

- Morning session n=400 (n=40 per school)
- Afternoon session n=400 (n=40 per school)

Based on sample size calculation, random selection of

In 10 schools n=800

- Morning session n=400 (n=40 per school)
- Afternoon session n=400 (n=40 per school)

Parental consent obtained

- Morning session 80%
- Afternoon session 94%

Parental consent obtained

- Morning session 82%
- Afternoon session 92%

**Not receiving emergency SFP (control)**

**Parent consent forms sent** 10 schools n= 3000

- Morning session n=1500 (n=150 per school)
- Afternoon session n=1500 (n=150 per school)

**Emergency SFP (intervention)**

**Quantitative data collection**

1. schools n= 782

Child Surveys with

- Morning session n=403 (Grade 4: n=156, grade 5: n=121, grade 6: n=126)
- Afternoon session n=379 (Grade 4: n=150, grade 5: n=133, grade 6: n=96)

**Qualitative data collection**

- Interviews with 12 school directors
- 3 focus group discussions with teachers and school staff (n=22)
- 3 focus group discussions with parents (n=29)

**Not receiving emergency SFP (control)**

**Quantitative data collection**

1. schools n= 800

Child Surveys with

- Morning session n=399 (Grade 4: n=141, grade 5: n=126, grade 6: n=132)
- Afternoon session n=401 (Grade 4: n=135, grade 5: n=135, grade 6: n=131)
